# Primary renal carcinoid tumors: Three case reports

**DOI:** 10.1097/MD.0000000000024714

**Published:** 2021-02-26

**Authors:** You Chen, Yuqing Shu, Laichang He, Kaifu Wu

**Affiliations:** Department of Radiology, The First Affiliated Hospital of Nanchang University.

**Keywords:** computed tomography, kindey, primary renal carcinoid tumors

## Abstract

**Rationale:**

Primary renal carcinoid tumors are very rare and only about 100 cases have been reported in the medical literature. There are even fewer articles on the imaging manifestations of primary renal carcinoid tumors.

**Patient concerns:**

We present 3 cases of patients with lumbago and hematuria. These were cases of primary renal carcinoid tumors with initial suspicion of suprarenal epithelioma.

**Diagnoses:**

Renal lesions were detected on abdomen computed tomography (CT) imaging. The 3 cases presented as solid/cystic solid renal mass with uniform or non-uniform density, calcification in the mass, and enhanced heterogeneity.

**Interventions:**

The laparoscopic partial/radical nephrectomy were performed.

**Outcomes:**

The postoperative histological diagnosis were primary renal carcinoid tumors. Case 2 been lost to follow-up in 3 patients, and the other 2 patients (Case 1 and 3) are still alive. Case 1 had intrahepatic metastases.

**Lessons:**

Primary renal carcinoid tumors mostly present as solid mass/cystic solid mass with calcification and delayed enhancement of heterogeneity on CT imaging, but the diagnosis depends on pathological diagnosis. Hence, raising awareness of the CT features of the rare tumor in the kidney may broaden the knowledge base of radiologists.

## 1 Introduction

Carcinoid tumors, which can arise in any organ, but are most found in bronchopulmonary and gastrointestinal system,^[1]^ and is extremely rare in kidney. There are just almost 100 cases of carcinoid tumors in kidney since the first case was reported in 1966.^[2–4]^ Most of the primary renal carcinoid tumors (RCT)are misdiagnosed due to lack of the specific clinical manifestations. Therefore, radiological evaluation of primary RCT can be challenging.

## 2 Case reports

### 2.1 Case 1

**Figure 1:**
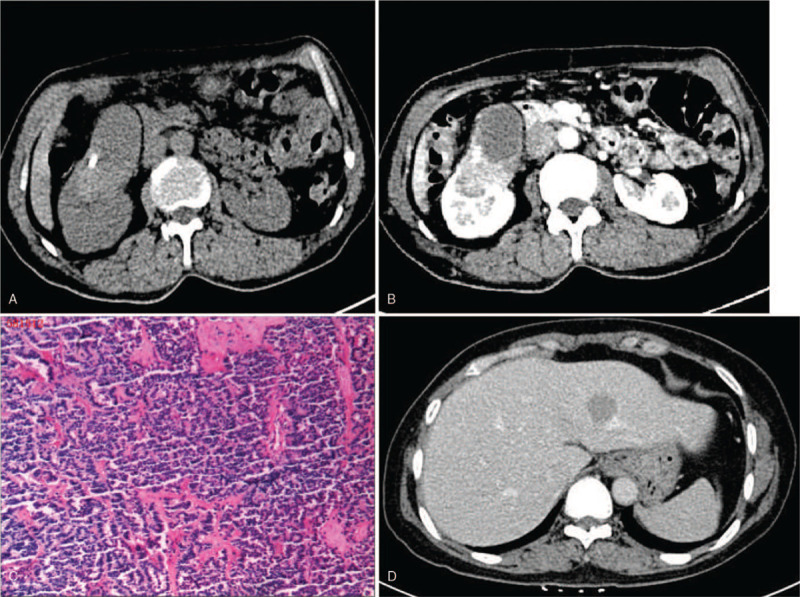
Case 1. Primary renal carcinoid tumor in a 59-year-old woman. A well-circumscribed mass and a heterogeneous enhancement pattern (1B) observed in the middle of the right kidney, with calcification and cystic degeneration in the mass (1A). Microscopic test revealed the tumor cells arranged in a sheet, trabecular or daisy-like shape, and calcium deposits was showed in the mass (1C). Intrahepatic metastasis was observed during follow-up (1D).

A 59-year-old female came to our hospital with stomachache and intermittent hematuria for a month in 2013. And his physical examination showed sputum pain in the right renal region. He denied the existence of other systemic diseases and family disease history at that time. And his urine analysis showed no positive finding, except for a large number of erythrocytes. Abdominal computed tomography (CT) revealed a well-defined mass in the right kidney with calcification in the mass, and the mass mixed cystic and solid components (Fig. 1A). The enhanced CT scan showed obvious enhancement of solid components of the mass, which was lower than renal parenchyma, and the CT values in arterial phase, venous phase and delayed phase were 89HU, 111HU, 93HU respectively when plain scan was 52HU. And the cystic part was not strengthened (Fig. 1B). Then he underwent laparoscopic partial nephrectomy in 2013. The resected mass in macroscopic pathological examination showed a grayish-yellow mass, which was 6 × 4 × 5 cm. And the mass was mixed cystic and solid. No obvious renal pelvis and ureteral involvement were observed. Microscopically, the tumor cells arranged in a sheet, trabecular or daisy-like shape. This mass, which had infarction rather than true tumor necrosis, had a mitotic rate of <2/10 high-power fields (HPF). And calcium deposits was showed in the mass (Fig. 1C). Immunohistochemical ex amination showed tumor cells were positive for synaptophysin while the chromogranin was negative. Both the microscopic and immunohistologic finding were consistent with the primary RCT diagnosis. And no any other metastases were showed after 12 months follow-up in 2014. The patient did not come to the hospital for follow-up examination during 2015 to 2017. But he was found with intrahepatic metastatic mass in 2018 (Fig. 1D), and the pathological results after hepatectomy were consistent with the diagnosis of metastatic carcinoid and the Ki-67 index>5%. The patient was lost to follow-up as he did not visit the hospital again.

### 2.2 Case 2

**Figure 2:**
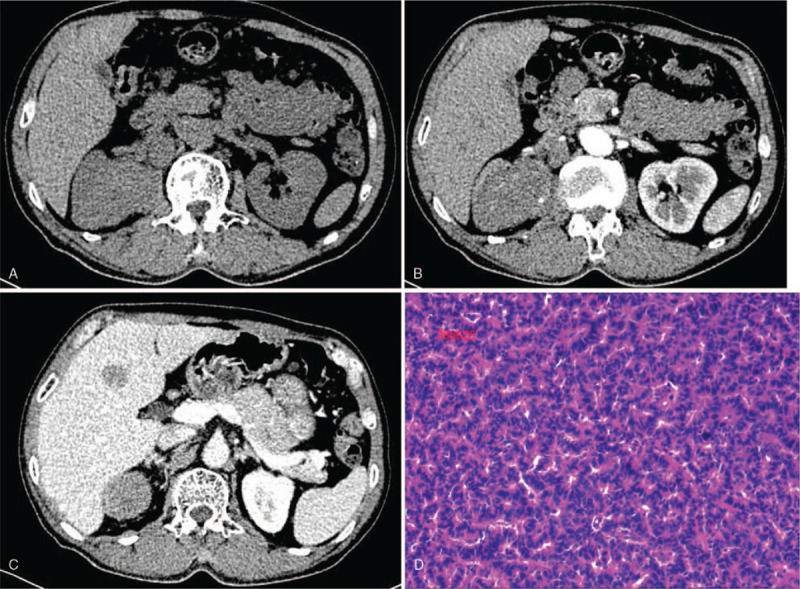
Case 2. Primary renal carcinoid tumor in a 66-year-old man. A partially calcified mass (2A) and a mild homogeneous enhancement pattern (2B) was observed in the right kidney. Hilar and retroperitoneal lymph node metastases (2A, 2B) and intrahepatic metastases (2C) were also observed. Microscopic examination revealed that the neoplastic cells arranged in irregular glandular or annular pattern with infiltrating growth, interstitial fibrosis, and angiogenesis.

A 66-year-old male presenting with hematuria in 2016. On abdominal CT, he was found to suffer a 6-cm-large partially calcified right renal mass, which was mild enhanced (Fig. 2A-B), and the CT values of solid components in arterial phase, venous phase and delayed phase were 45HU,59HU,67HU respectively while plain scan showed 42HU. CT scan also showed that he had lymphadenopathy in renal hilum and retroperitoneal. Besides, intrahepatic metastases was also observed (Fig. 2C). Laparoscopic radical nephrectomy was subsequently performed. The macroscopic specimen of the surgical specimen showed a unifocal 6.0 cm in diameter mass. Microscopic examination revealed that the tumor cells were arranged in irregular glandular or annular arrangement, with infiltrating growth, interstitial fibrous tissue hyperplasia with hyaline degeneration, vascular hyperplasia, dilation and hyperemia, and patchy necrosis was seen in the mass (Fig. 2D). Immunohistochemical tests showed that the mass was positive for chromogranin and synaptophysin while CD56 was negative. The Ki-67 index was 10%. Both microscopic and immunohistologic findings were complied with renal carcinoma. CT imaging 8 months after surgery showed metastatic progressively growing lymphadenopathy and liver lesions. The patient was lost to follow-up as he did not visit the hospital again.

### 2.3 Case 3

**Figure 3:**
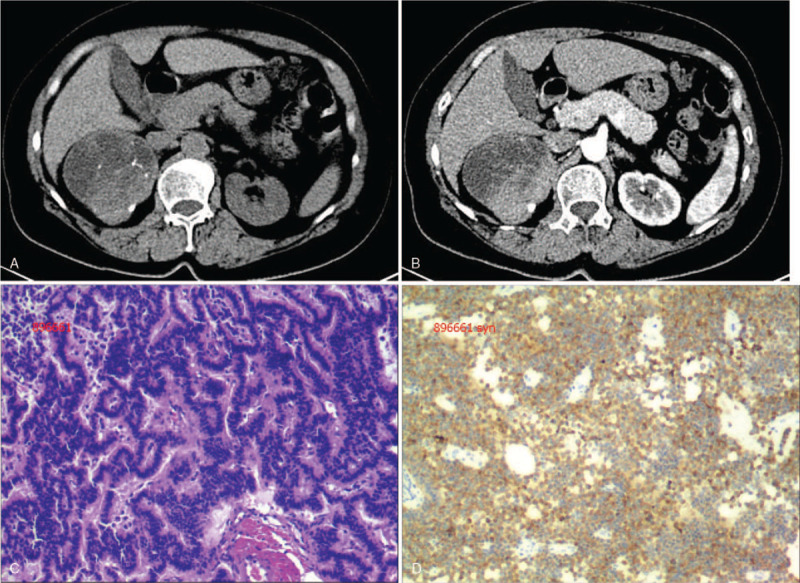
Case 3. Primary renal carcinoid tumor in a 58-year-old woman. Partial calcification and necrosis in right renal mass (3A) and a heterogeneous enhancement pattern (3B)was observed in the right kidney. Microscopic evaluation showed that the tumor cells were arranged in a papillary or irregular glandular tube (3C). Immunohistochemical test showed the renal tumor was positive for synaptophysin (3D).

A 58-year-old female, presenting with intermittent hematuria and flank pain. In 2018, she was found to have a 8.0 cm partially calcified and necrosis right renal mass without any lymphadenopathy and metastases visualized (Fig. 3A). The enhanced CT revealed an obvious contrast enhancement of solid components of the mass in right renalwhile the cystic part was not strengthened (Fig. 3B), and the CT values of solid components in arterial phase, venous phase and delayed phase were 61HU, 90HU, 86HU respectively while plain scan showed 42HU. Then a right radical nephrectomy was performed. Macroscopic evaluation showed a 8 × 6 × 7 cm kidney tumor. Microscopic evaluation showed that the tumor cells were arranged in a papillary or irregular glandular tube and negative margin of resection. The mass had the necrosis (Fig. 3C). On immunohistochemical test, the renal tumor was positive for synaptophysin (Fig. 3D) while chromogranin was negative. There is no sign of recurrence or metastasis till now after surgery.

Our study was approved by the Ethics Committee of the First Affiliated Hospital of Nanchang University, and the family members of the patients have provided informed consent for the publication of the case.

## 3 Discussion

RCT are considered to be a rare disease for lacking neuroendocrine cells in kidneys, and they also lack specific clinical manifestations. Sometimes, primary RCT are asymptomatic because of the slow growth of tumor.^[3]^ In addition, some of the symptoms of RCT include abdominal or lumbago, hematuria, constipation, frequent urination, fever and a loss of weight.^[2,4]^ The vasoactive substances (serotonin) produced by tumors cause clinical manifestations of carcinoid syndrome such as blushing or diarrhea.^[5,6]^ About 13% RCT patients show carcinoid syndrome,^[5]^ but none of our cases showed this clinical manifestation. The pathogenesis of primary RCT are not clear, there are many hypotheses about the tumors^[4,7,8]^:

(1) some scholars have proposed that tumor cells originate from endogenous neuroendocrine cells, or microendocrine-paracrine or pancreatic cells;
(2) it has also been suggested that tumor cells originate from intestinal metaplasia of glomerular epithelial cells caused by chronic infection or inflammation;
(3) 3)and some other scholars believed that tumor cells were generated by activating gene sequences of neuroendocrine cells or primitive pluripotent cell lines;
(4) besides, chromosomal aberrations may induce predifferentiation of renal tumors, including renal carcinoid tumors.

The incidence of right kidney carcinoid is higher than that of left kidney, with no gender difference, but it is related to horseshoe kidney, teratoma and polycystic kidney. RCT associated with horseshoe kidneys, usually occur in men.^[4,7,9]^ All our three cases showed occurrence in the right kidney, but none of them had such lesions such as horseshoe kidney, teratoma or polycystic kidney.

Primary renal carcinoid tumor usually presents as a single solid or cystic solid mass, with isolated and irregular marginal lobes, destruction of renal parenchyma, internal hemorrhage, necrosis and cystic changes. Cystic degeneration may be unilocular or multilocular. And hemorrhagic necrosis of the mass is associated with neovascularization, rapid tumor growth, or compression of the blood supply artery in the lesion area.^[7]^ About 25% of the patients have calcification,^[7]^ which may be related to the long-term growth of the tumor or/and the presence of teratoma-like changes. To some extent, it is seems to indicate a better prognosis of the tumors. Ultrasound findings in renal carcinoid tumors demonstrate a hyper echoic mass with an incomplete hypo echoic or anechoic thin rim or halo and central or peripheral calcifications, but it is hard to diagnosis alone because the findings are sometimes similar to findings in small renal cell carcinoma. And CT findings usually demonstrate a realistic/cystic solid mass with calcification and mild contrast enhancement of solid components, which could be used for diagnosis and differential diagnosis.^[2]^ All our 3 cases of primary RCT were found with calcification in the lesion. On CT, they were heterogeneously enhanced but less than the renal cortex in corticomedullary phase.^[3]^ There were 2 of Our cases which showed obvious enhancement in the solid part, no enhancement in the cystic part, and the degree of enhancement was lower than that in the renal cortex at the same period, and the parenchymal and excretory phase showed delayed enhancement. However, some literature showed that the primary renal carcinoid was a hypoxic tumor with no enhancement or mild enhancement.^[2,10]^ 1 of our cases presented mild enhancement with slight delayed enhancement in the parenchymal and excretory phases.

Our cases are unique because carcinoids occur in the kidneys, and all presented as cystic-solid masses, among which 2 cases had obvious enhancement of solid components and 1 case had mild enhancement of solid components. RCT are often misdiagnosed as kidney cancer, because of the similar clinical and radiological features. So primary RCT needs to be differentiated from kidney cancer. Kidney cancer is the most common malignant tumor of the kidney, often presenting as a lump of the kidney on one side, with a slightly lower density on plain scan and obvious enhancement, presenting as “fast in and fast out” on CT. Kidney cancer is a tumor with rich blood supply, often accompanied by hemorrhage, necrosis and cystic degeneration, but rarely with calcification.

At present, surgery is the most important treatment of kidney carcinoid tumors. The surgical methods include laparoscopic partial/radical nephrectomy.^[3]^ Patients with regional lymph node metastasis will perform lymph node dissection at the same time. Carcinoids grow slowly and will have a long course of disease. They are considered to be low-grade malignant tumors. However, because they are prone to infiltration and metastasis, clinical outcomes are difficult to predict. So regular follow-up is needed after surgery.

In conclusion, carcinoid need to be considered when a renal cystic solid mass is observed with uniform or non-uniform density, calcification in the mass, obvious/slight enhancement in arterial phase of enhanced scan, gradual enhancement in vein and delayed phase, especially in the presence of cardiovascular or gastrointestinal symptoms.

## Author contributions

**Data curation:** Kaifu Wu.

**Funding acquisition:** Laichang He.

**Writing – original draft:** Yuqing Shu.

**Writing – review & editing:** You Chen.
